# Modelling Visual Neglect: Computational Insights into Conscious Perception

**DOI:** 10.1371/journal.pone.0011128

**Published:** 2010-06-15

**Authors:** Linda J. Lanyon, Susan L. Denham

**Affiliations:** 1 Human Vision and Eye Movement Laboratory, Departments of Ophthalmology and Visual Science, Medicine (Neurology), Psychology, University of British Columbia, Vancouver, British Columbia, Canada; 2 School of Psychology, Centre for Robotics and Neural Systems, University of Plymouth, Plymouth, United Kingdom; University College London, United Kingdom

## Abstract

**Background:**

Visual neglect is an attentional deficit typically resulting from parietal cortex lesion and sometimes frontal lesion. Patients fail to attend to objects and events in the visual hemifield contralateral to their lesion during visual search.

**Methodology/Principal Finding:**

The aim of this work was to examine the effects of parietal and frontal lesion in an existing computational model of visual attention and search and simulate visual search behaviour under lesion conditions. We find that unilateral parietal lesion in this model leads to symptoms of visual neglect in simulated search scan paths, including an inhibition of return (IOR) deficit, while frontal lesion leads to milder neglect and to more severe deficits in IOR and perseveration in the scan path. During simulations of search under unilateral parietal lesion, the model's extrastriate ventral stream area exhibits lower activity for stimuli in the neglected hemifield compared to that for stimuli in the normally perceived hemifield. This could represent a computational correlate of differences observed in neuroimaging for unconscious versus conscious perception following parietal lesion.

**Conclusions/Significance:**

Our results lead to the prediction, supported by effective connectivity evidence, that connections between the dorsal and ventral visual streams may be an important factor in the explanation of perceptual deficits in parietal lesion patients and of conscious perception in general.

## Introduction

**Figure 1:**
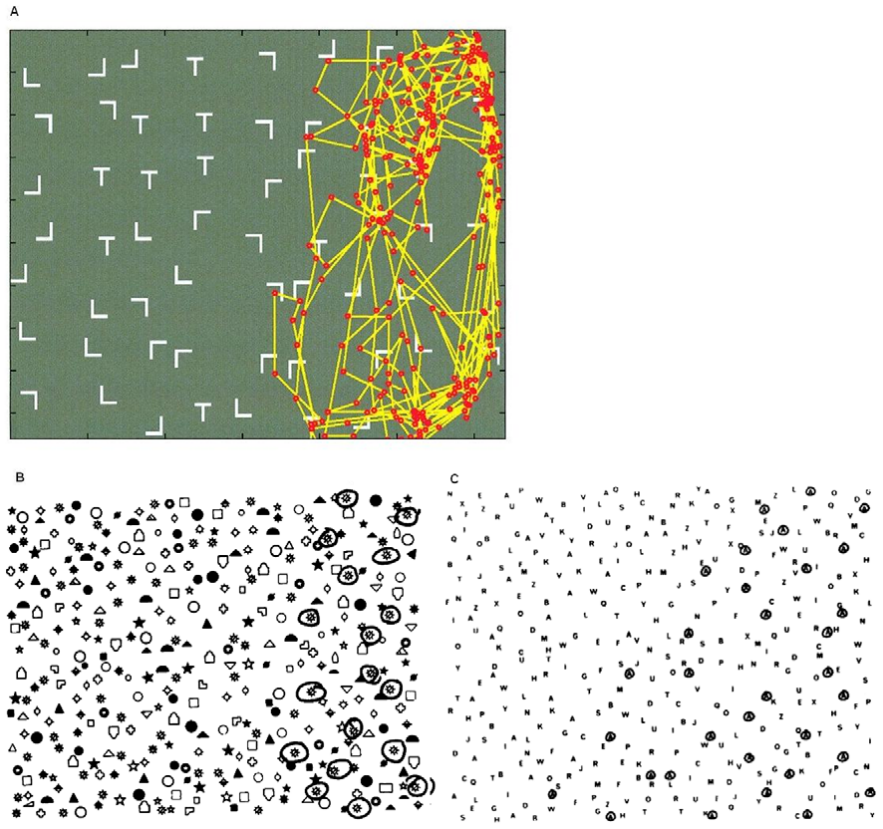
Examples from neglect patients. a. Example scan path from patient G.K. who has infarction of the **right inferior parietal lobe but has sparing of the frontal lobe** [1]. The search task was to find letter Ts amongst distractor Ls. There is profound neglect of the left side of the array and many re-fixations on the right. Figure from Husain et al. [1] ‘Impaired spatial working memory across saccades contributes to abnormal search in parietal neglect’ Brain (2001), 124, 941–952, by permission of Oxford University Press. b. Visual targets successfully cancelled during a cancellation task involving random shapes by Weintraub and Mesulam [52]. This patient has a **large right parieto-temporal lesion** and dramatically neglects the left hemifield. Reproduced from ‘Visual hemispatial inattention: stimulus parameters and exploratory strategies’ Weintraub and Mesulam [52] Journal of Neurology, Neurosurgery and Psychiatry, 51(12), 1481–8, (1988) with permission from BMJ Publishing Group Ltd. c. Visual targets successfully cancelled during a cancellation task involving randomly positioned letters by Weintraub and Mesulam [52]. This patient has a **right superior frontal infarct** and neglects the left hemifield. Reproduced from ‘Visual hemispatial inattention: stimulus parameters and exploratory strategies’ Weintraub and Mesulam [52] Journal of Neurology, Neurosurgery and Psychiatry, 51(12), 1481–8, (1988) with permission from BMJ Publishing Group Ltd.

Visual neglect (also referred to as “unilateral neglect”, “hemispatial neglect” or “hemineglect”) can result from a lesion typically to the posterior parietal cortex [1]. It is also sometimes associated with frontal lobe lesions [2], [3] or lesion of the thalamus [4]. These neurological patients suffer an inability to notice objects and events in the hemifield contralateral to their lesion. Deficits in perception appear to be related to attentional factors, rather than being purely sensory, because problems are often context dependent. Although neglect patients are able to make saccades in the contralesional direction during visual search, their fixations tend to be concentrated in the ipsilesional hemifield [1], [5]. Examples of such a scan path and patient target detection performance are shown in figure 1. Patients have a tendency to frequently re-fixate targets and, in parietal cases, re-fixation rates increase with time since first fixating a location [1]. Neglect patients whose lesions include frontal cortex are prone to motor perseveration during paper-and-pen line cancellation tasks, repeatedly marking the same line and seeming less able than normals to move their attention away [6]. In particular, orbitofrontal lesion has been linked to increased re-fixation in search scan paths [7]. Unlike those associated with parietal injury, re-fixation errors following frontal lesion do not appear to increase with time since first fixating a location [1].

We have previously presented a computational model of visual attention and search [8], [9], [10], [11] that is based on and constrained by neurobiology. The cellular properties of the model are based on evidence from monkey electrophysiology and the model has been used to accurately simulate activity observed in monkey single cell studies of visual attention, such as those performed by Chelazzi et al [12], [13], [14]. On the basis of this biological constraint at the cellular level of the model, the overall system produces eye movement behaviours similar to those observed in normal human and monkey psychophysical studies [8], for example those reported by Motter and Belky [15] during visual search for a feature conjunction target. Hence, the model provides a platform for testing the link between neuronal activity and behaviour, and the effect of lesions, because it captures visual attentional behaviours at both the single cell and behavioural levels.

Our aim for the current work was to examine the effects of parietal lesion in the model on simulated visual search behaviour. Under normal conditions the model produces search scan paths that thoroughly investigate a scene and exhibit biologically-realistic inhibition of return (IOR), a bias against returning to locations that have been already inspected [16], [17], [18]. Would a parietal lesion result in altered scan paths during visual search? We will describe how the model simulates scan paths under lesion conditions that resemble those produced by parietal patients suffering symptoms of visual neglect, including the scan path neglecting the contralesional hemifield and exhibiting an increased rate of re-fixation. We also investigated the effect of lesion to the frontal bias to parietal cortex, on scan paths and re-fixation rates. Frontal damage has been linked to neglect behaviours [2], [3], [19], deficient IOR behaviour [7] and perseveration [6]. Our frontal bias is an external signal to the system that mediates IOR and is represented in scene-based coordinates. Would a unilateral lesion to an area representing a scene-based frame of reference lead to the same symptoms of neglect as lesioning a retinotopic parietal area? We will describe how impairment of this model's frontal bias leads to symptoms of neglect, deficits in IOR and, hence, perseveration.

The extrastriate ventral stream has been linked with the conscious perception of stimuli [20], [21], [22]. In our simulations this region remained free from lesion and represented stimuli across the scene. However, we found differences in levels of extrastriate activity when performing simulations with parietal lesion: neglected stimuli were represented with lower levels of activity than non-neglected stimuli. We will discuss reasons for this effect in our model in relation to findings in neuroimaging. Such differences in levels of activity in our model could represent a computational correlate of conscious versus unconscious perception following parietal lesion.

## Materials and Methods

### The Existing Model

**Figure 2:**
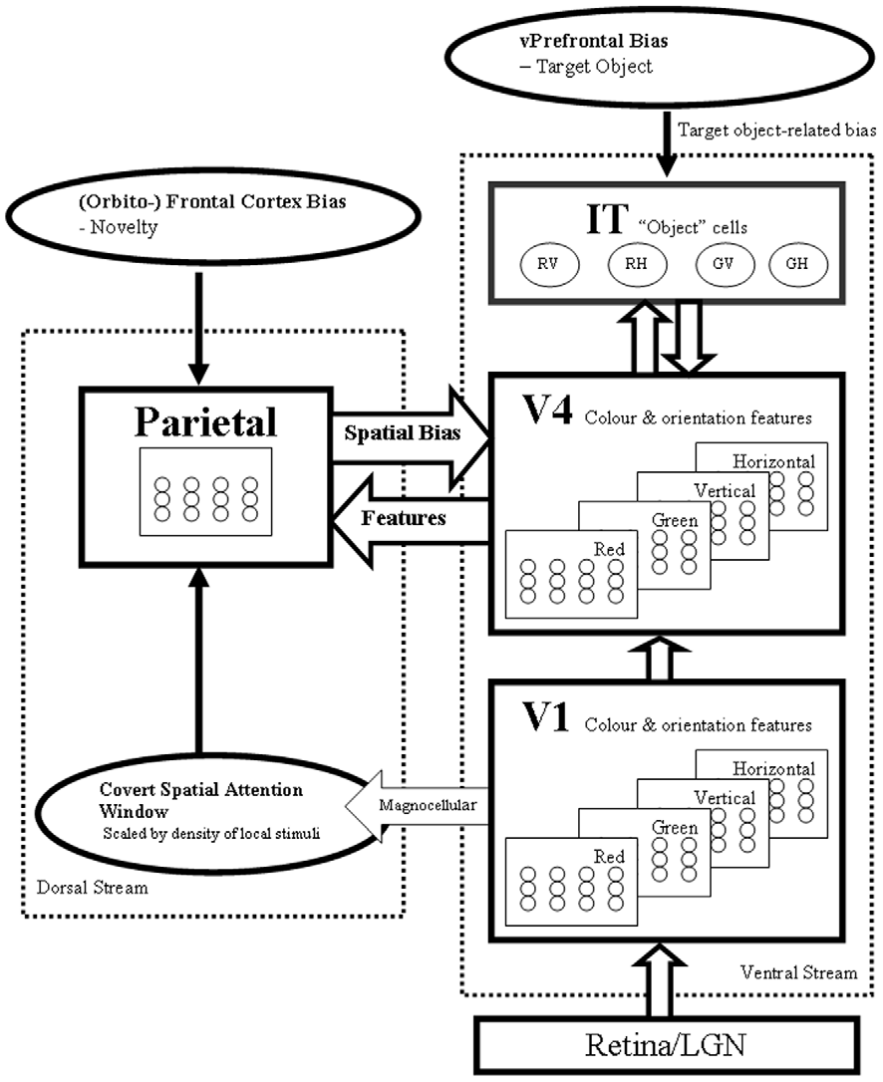
Model Architecture. Schematic of the modules within the system. Note that the actual number of cells in V1, V4 and the parietal module are more than shown, and inhibitory interneurons are not shown. For further details see Lanyon and Denham [8], [11].

Our computational model, described fully by Lanyon and Denham [8], [11], was inspired by seminal biased competition modelling by Deco [23], [24], and is depicted in figure 2. In addition to the overt shifts of attention previously modelling, we include here an extension to the model to allow covert shifts of attention where fixation is maintained at the centre of the display. Each module consists of many neurons whose activity is updated at each time step, according to inputs from other neurons in the same or other modules and bias signals that originate from brain regions external to the system (such as a frontal bias relating to the search target). According to a widely accepted neurobiological model that suggests cortical visual processing is performed in two main streams [25], [26], our model consists of a ventral and a dorsal stream. Communication across the streams is also a feature of our model and recent empirical evidence suggests that the two streams are less separable than previously thought [27].

In the model's ventral stream, colour and orientation features are processed in a feed-forward hierarchical fashion from the retina through visual area V1 to area V4 and then the anterior inferior temporal area (IT). Retinal ganglion broad-band cells perform simple centre-surround processing and retinal concentric single opponent cells process colour information, as we have described previously [8], [11]. Area V2 is not included in the model on the basis that we are using very simple stimulus sets and similar feature detection processes would be performed in a V2 module as those described for V1 and V4. Areas V1 and V4 encode features in a retinotopic manner with receptive field sizes being biologically realistic and larger in V4 than V1 (see [8] for further detail). These areas contain a retinotopic feature map for each feature represented (red & green colours and vertical & horizontal orientations). Area V4 feeds forward to area IT, which encodes invariant object representations. Within the ventral stream, biased competition operates between different features and different objects. During visual search, object-based attention results from a bias from frontal cortex (possibly ventral prefrontal area 46), which represents the search target, fed back to influence the competition between objects in IT. IT feeds back to V4 so that, as the target object wins the competition within IT, the feedback bias results in target features being enhanced and non-target features being suppressed in parallel across V4. In order to shift attention covertly, a spatial attentional focus is fed through the parietal module to area V4, resulting in spatial attention effects in that area. So, in area V4, both spatial and object-based attentional effects emerge from the dynamics of the system.

The model's dorsal stream consists of a retinotopically organised parietal module where competition operates between different retinotopic spatial locations. The location becoming most active wins the competition to attract attention and, hence, becomes the new focus of attention. The parietal module receives inputs from V4 so that, as V4 begins to represent the location of possible target features most strongly, these locations receive a favourable bias within the parietal module. Hence, possible target locations become enhanced and locations not containing target features become suppressed. The parietal module, therefore, acts as an indicator of behavioural relevance: it is known that the monkey lateral intraparietal area represents the behavioural significance of stimuli [28].

The parietal module receives another external bias that reflects the novelty of locations in the scene and, hence, their potential reward (given that locations already visited have low reward for revisiting). This scene-based (or possibly body-based) representation could form part of a high-level search strategy that is controlled by frontal cortex, and might be represented in orbitofrontal cortex because of the links of this area with reward [7], [29]. In our model, this “novelty map”, which is the size of the complete image, indicates whether locations in the scene have been previously inspected and mediates IOR in the search scan path by contributing an excitatory bias to the competition in the parietal module. The map is expressed in a scene-based coordinate system and is transformed to a retinal coordinate system within the parietal module. After a location has been visited novelty in that region is reduced such that there is a gradient of inhibition around the previously attended location [17]. Novelty then takes some time to recover so that attention is inhibited from several of the most recently visited locations [30]. Hence, a recently visited location will have a negative novelty value and values in the local vicinity will increase in a gaussian fashion to neutral (zero) novelty over the extent of an Attention Window (AW), which is scaled based on stimulus density as described by [31]. Areas of the scene that have yet to be inspected will have high (positive) novelty value. Under normal conditions the novelty bias to the competition in the parietal module causes the scan path to be effective in exploring the scene and not perseverating in any particular area [8].

Since lesion of the parietal module and the frontal bias signal is the focus of this work, we now provide the mathematical description of this part of the model. Other aspects are described in Appendix S1. The pyramidal cell assemblies in the parietal module evolve according to the following dynamics:
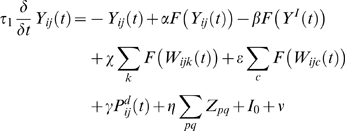
(1)where:

α is the weight of excitatory input from other cells in the pool, set to 0.95

β is the weight of inhibitory input, set to 1

W_ijk_ is the orientation input from V4 for orientation k, at location (i,j)

χ is the weight of V4 orientation inputs, normally set to 0.8 (see [8] for examination of relative weighting)

W_ijc_ is the colour input from V4 for colour c, at location (i,j)

ε is the weight of V4 colour inputs, normally set to 4


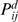
 is the spatial AW bias injected directly into this pool when there is a requirement to attend to this spatial location

γ is the weight of the spatial AW bias, set to 2.5

Z_pq_ is the bias from area pq of the novelty map (which is the size of the original image, N). Area pq represents the size of the parietal receptive field. (See [8] for more information about the novelty bias)

η is the weight of the novelty bias, normally set to 0.0009

I_0_ is a background current injected in the pool, set to 0.25

ν is additive noise, which is randomly selected from a uniform distribution on the interval (0,0.1)

The dynamic behaviour of the associated inhibitory pool in LIP, providing competition between locations, is given by:
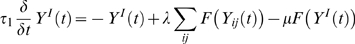
(2)where:

λ is the weight of pyramidal cell assembly input, set to 1

μ is the weight of inhibitory interneuron input, set to 1

### Simulated Lesions

Simulating a parietal cortex lesion in this model is the focus of this work. Our aim was to apply a unilateral lesion to the parietal module in order to simulate a right hemisphere parietal lesion, which has been associated with severe symptoms of visual neglect in search scan paths [32], [33]. The lesion was simulated by reducing the activity in the left half of the parietal module, in order to simulate a right hemisphere lesion since crossover of the optic radiations at the optic chiasm is not specifically represented in this model. In different lesion simulations the activity was either set to zero across the complete hemifield (a *complete hemi-lesion*) creating a step-function of impairment, or was reduced in the hemifield by an amount that increased in a gradient fashion from the centre of the visual field towards the periphery (a *gradient lesion*). Some authors have argued that neglect manifests as a gradient across the visual field [33] and parietal lesions have been simulated elsewhere with a gradient of impairment [34], [35], [36]. The method we use is a simple linear gradient in which the amount of deficit was varied more or less steeply towards the contralesional visual periphery. The gradient of impairment started at values ranging from 1 to 100% at the far left of the hemifield and reduced to zero at the centre of the visual field. Search scan path behaviours were compared at these different gradients of impairment.

A complete unilateral parietal hemi-lesion is given by:
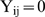
(3)where:

i ranges from 1 to m, m being the number of rows of neurons in the parietal module

**j ranges from 1 to n/2**, n being the number of columns of neurons in the parietal module, an index of the lateral position of the neuron

A parietal gradient lesion is given by:
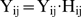
(4)where:

i and j range as above

H is the gradient of impairment matrix in which the values in each row increase linearly left to right from starting value x, the maximum impairment, to 1, no impairment.

In addition to having search scan paths which typically favour the ipsilesional hemifield, neglect patients also have impaired spatial memory of locations previously visited [1] and demonstrate perseverance, particularly when frontal cortex is involved [6]. We simulated lesions to the frontal representation of novelty, which is represented in a scene-based frame of reference, in order to examine the effect on scan paths of both a unilateral and bilateral frontal novelty/reward lesion.

A frontal lesion is given by:
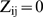
(5)where:

i ranges from 1 to N, N being the size of the original image

j ranges from 1 to N/2, for a unilateral lesion; j ranges from 1 to N, for a bilateral lesion

### Attentional Scanning

We have previously described the model in active visual search mode i.e. using overt attention with eye movements [8]. The model's retina processed a portion of the entire scene centred at the current fixation point and the cortical modules processed only what was present within the current retinal view. This presents an issue when simulating a parietal lesion since, unlike the real continuous world, a simulated scene has boundaries. The parietal module determines the next location for fixation. If fixation lands at a location very close to the right edge of the scene, and the left of the parietal module is ineffective due to lesion, the system may be unable to find a new location to which to move its focus of attention. This happens because the intact right half of the parietal module extends beyond the scene and does not receive any bottom-up featural information. (Note, the normal practice of computationally extending/replicating the scene beyond its boundaries does not help to move the scan path back into the scene in this situation.) For this reason, we extended the model to operate using covert attention in which fixation is maintained at the centre of the scene and the entire scene is present on the retina at all times but the focus of spatial attention moves. Most leading computational models of visual attention (for example, [23], [37], [38]) and neglect [34], [35], [36] tend to use covert rather than overt attentional movements so that the entire scene is processed for each location that is attended. However, the use of overt attention with eye movements is more biologically realistic, such that only the portion of the scene present on the retina is processed. We present simulations using both approaches. For our overt simulations we also show the effect of applying a “top-down” search strategy where a small leftward saccade is made to shift fixation back into the scene in the situation where fixation lands at the rightmost border of the scene. Note, although reports of laterality bias in patients' saccades vary, some show as many leftward as rightward saccades in the patient's normal hemifield [1], [39].

## Results

### Unilateral Parietal Lesion - Scan Path

**Figure 3:**
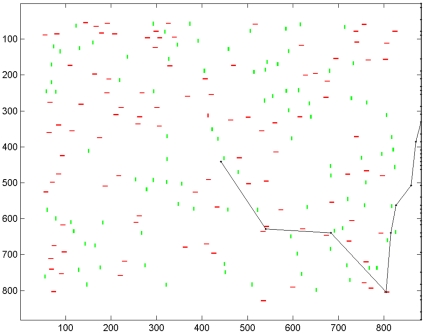
Simulated scan path following parietal lesion without leftward reset. A typical scan path obtained when the parietal module is unilaterally lesioned under overt attention and no leftward reset back into the scene is present at the rightmost border. The first fixation is placed at the centre of the image. From there the scan path is attracted to the right and becomes unable to re-orient away from the rightmost border of the scene.

**Figure 4:**
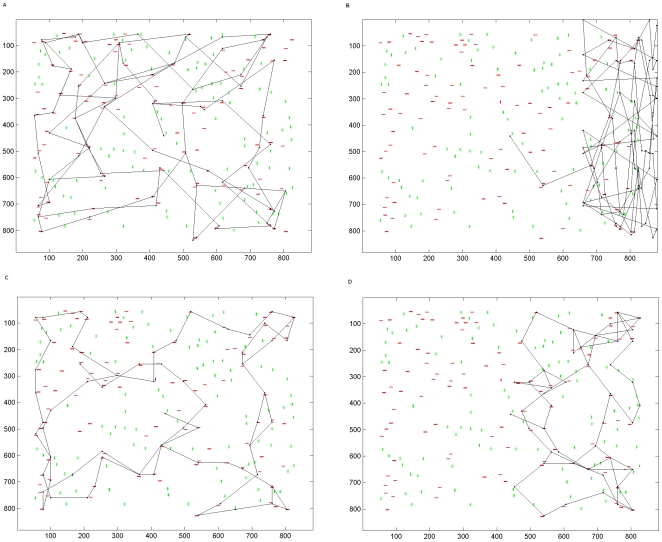
Simulated scan paths following parietal lesion. a. A scan path obtained using the intact version of the model under overt attention. The target object is a red bar; hence most fixations land near red objects rather than near green objects or in blank regions of the scene. b. Scan path produced from overt attention when the parietal module is unilaterally lesioned. Severe symptoms of hemineglect are present in the scan path. This shows that visual hemineglect is produced when the parietal region that encodes stimuli in a retinotopic frame of reference is unilaterally lesioned and overt eye movements are made. This figure may be compared to the parietal patient behaviours shown in figure 1a and b. c. Attentional scanning movements produced by the intact model using covert attention. d. Symptoms of visual neglect in covert scanning produced when the model's parietal module was unilaterally lesioned. This shows that visual hemineglect is produced when the parietal region that encodes stimuli in a retinotopic frame of reference is unilaterally lesioned and covert attention is deployed. This figure may be compared to the parietal patient behaviours shown in figure 1a and b.

Scan paths exhibited complete neglect of the left hemifield of the visual display when the parietal module was unilaterally lesioned. This occurred for both covert and overt simulations. For reasons described above, scan paths tended to become stuck at the rightmost border of the scene during overt scanning; an example is shown in figure 3. Attention was attracted to the right hemifield but was unable to re-orient away from the rightmost border of the scene. If the strategy of a reset saccade to move left back into the scene from the rightmost border was adopted then attention was able to re-orient within the right hemifield but the left was severely neglected, as shown in figure 4b, in comparison to the scan path produced by the intact model in figure 4a. Similarly, under covert scanning, the scan path neglects the left hemifield when the parietal module is lesioned but not when it is intact, as shown in figure 4 c and d. This demonstrates that lesion of the retinotopically organised parietal module results in **neglect of the left hemifield whether or not the eyes are moved**. Neglect occurs despite locations in the left hemifield having high novelty value: the potential novelty and reward of these locations is not taken into account by the attention shifting decision process because of the parietal lesion. Throughout this results section we show simulations in which the initial fixation is at the centre of the display, in order to match typical starting points in many psychophysical studies. However, the normal and pathological scan path effects are robust and not dependant on initial fixation location.

### Gradient Parietal Lesion - Scan Path

**Figure 5:**
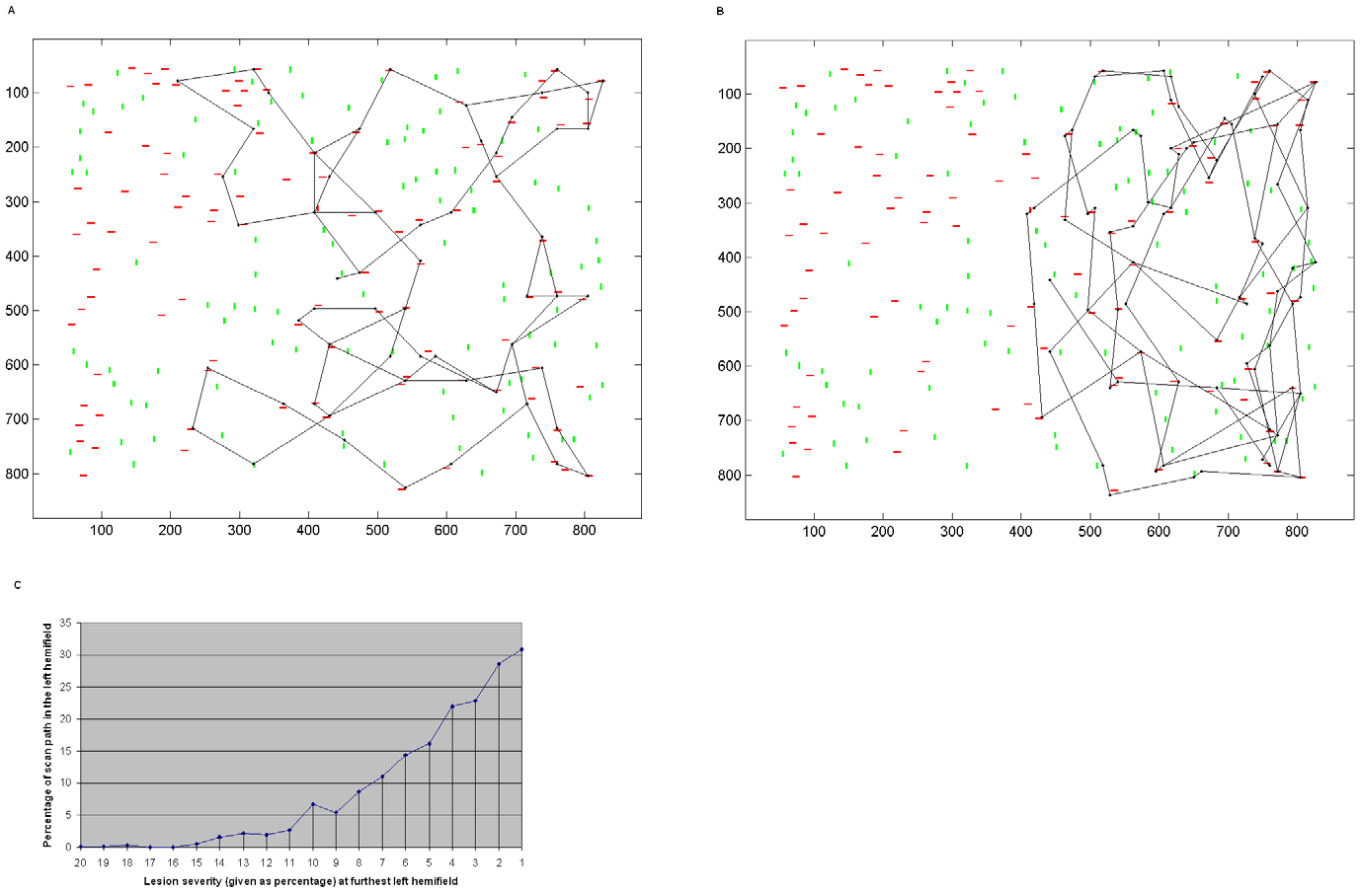
Attentional capture by the left hemifield when there is a gradient of impairment. a. An example of covert scanning under a gradient lesion when the parietal module was lesioned to reflect a gradient of impairment. The impairment is strongest at the left and improves towards the centre of the left hemifield. At the leftmost periphery the associated parietal neurons are 7% impaired. Across the left hemisphere impairment decreases in a linear fashion so that neurons at the centre and those in the right hemifield are unimpaired. Whilst most red (target colour) stimuli are attended in the right hemifield and the more central region of the left hemifield, the impairment is greatest towards the far left and stimuli here are neglected. b. The same simulation as (a) but using overt attention. c. Shows that attention is more likely to be captured by stimuli in the left hemifield when the gradient of impairment is less steep, i.e. the effect of the lesion is less extensive. Mean percentages from 10 separate simulations, each containing 100 fixations and using covert attention, are shown. Effects saturate (i.e. the hemifield is completely neglected) at 20% lesion at the far left of the hemifield.

When the simulated lesion incorporated a gradient of impairment, the left hemifield was again neglected (see example in figure 5a,b) but the extent to which the scan path entered the neglected hemifield was determined by the severity of the lesion i.e. the steepness of the gradient. Figure 5c shows this relationship. When the gradient was less steep more central regions of the left hemifield could be attended but regions that were more peripheral tended not to be attended. Hence, neglect of stimuli that are further to the contralesional visual periphery was more severe than for those closer to the centre of the visual field.

### Parietal Lesion – Effect on Cortical Activity

**Figure 6:**
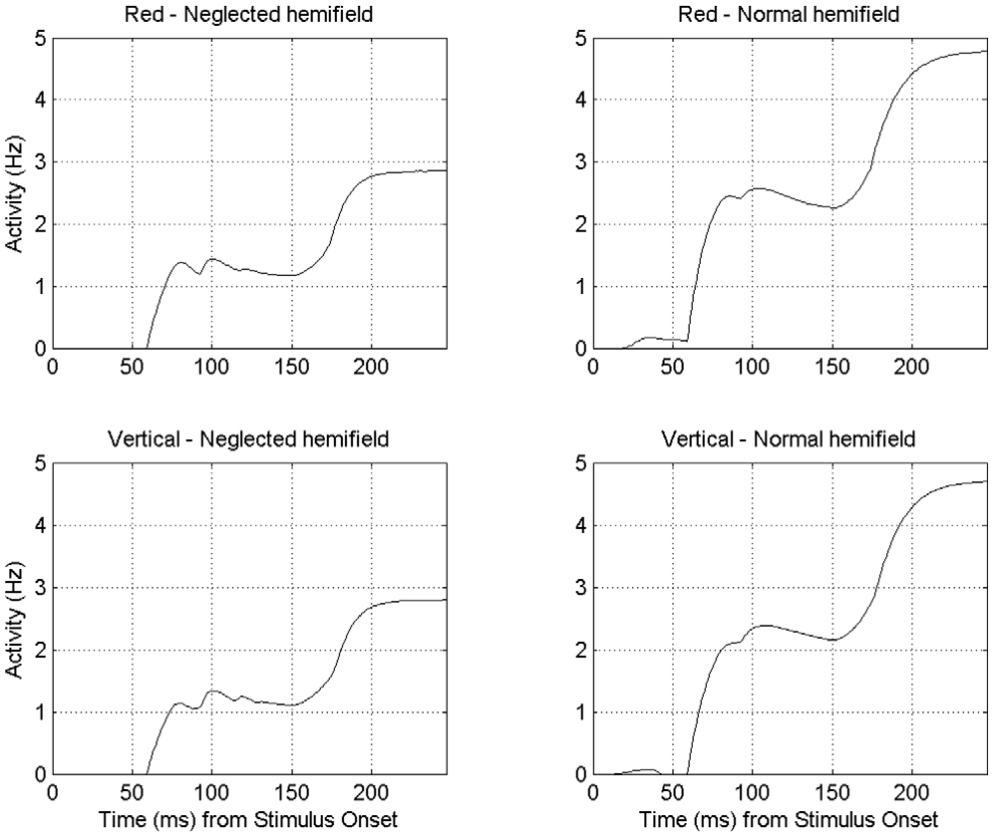
Effect of Parietal Lesion On V4 Activity. When presented with a scene containing a single red vertical bar in each hemifield, the responses of V4 cells that are selective for red or vertical features are shown. Responses from green and vertical selective cells are not shown since these were near baseline, due to lack of relevant stimuli in the scene. The plots on the left relate to cells that have receptive fields positioned left of the vertical meridian, in the neglected hemifield. The plots on the right are for cells with receptive fields in the normal right hemifield. Cells selective for the same feature have the same response properties except the position of their receptive fields. Activity for the stimulus in the neglected hemifield is reduced compared to that for the identical stimulus in the normal hemifield.

Although the model's ventral stream was completely intact, levels of activity in the ipsilesional side of V4 were affected by the parietal lesion. Activity for stimuli in the neglected left hemifield was much reduced compared to that in the right hemifield despite both hemispheres in V4 being free of lesion. Figure 6 shows this effect with a direct comparison of the activity elicited in V4 in response to two identical stimuli presented each in one hemifield. A single red vertical bar was presented either side of the vertical meridian and the responses of two V4 neurons whose receptive fields contained each stimulus were recorded. Figure 6 shows that, in response to the same stimulus, cells representing locations in the neglected left hemifield had lower activity than cells representing the normal right hemifield. We will discuss the biological significance of this in the discussion.

### Frontal Lesion - Scan Path

**Figure 7:**
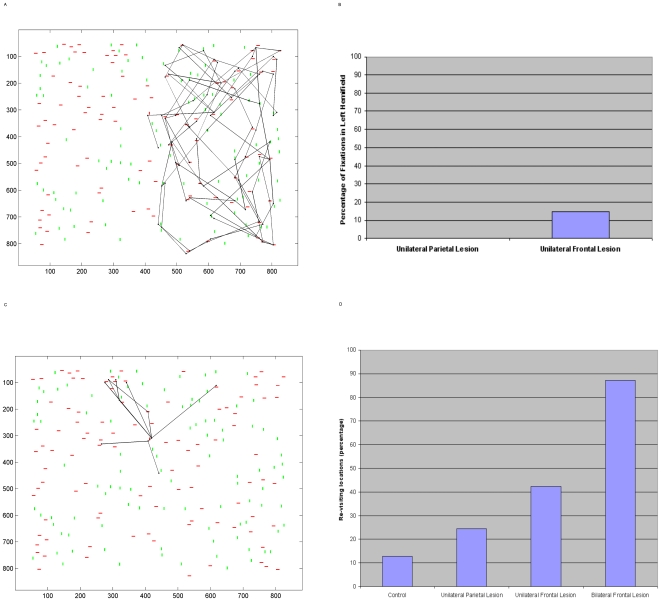
Frontal Lesion and Re-visiting Locations in the Scan Path. a. When the frontal module is unilaterally lesioned, symptoms of neglect are present in the scan path (overt attention shown here). This shows that a lesion in an area representing stimuli in a scene(or body)–based frame of reference can produce symptoms of neglect. This figure may be compared to the frontal neglect patient behaviour shown in figure 1c. b. A comparison of the percentage of fixations in the left hemifield following unilateral parietal versus unilateral frontal lesion in this model. The plot shows the mean percentages from 10 separate simulations, each containing 50 shifts of covert attention (values were similar under overt attention). Although the left hemifield is typically neglected following frontal lesion, some fixations are placed in this hemifield. Hence, compared to that following parietal lesion, neglect is less severe with frontal lesion in this model. c. When the frontal module is completely lesioned the scan path has difficulty exploring the scene and perseverates in one area. An overt attention simulation is shown here but similar effects are produced under conditions of covert attention. This is due to the lack of novelty bias in the system. d. Shows the effect of lesion on re-visiting of locations under covert attention. Locations are more frequently re-visited under conditions of parietal lesion than in normal conditions. However, lesion of the frontal bias in our model causes the greatest increase in re-visiting/re-fixation. The plot shows the mean percentages of re-visited locations from 10 separate simulations, each containing 50 shifts of attention.

The model's frontal bias to parietal cortex represents a scene-based encoding that indicates the novelty of searched locations in the scene. This bias mediates IOR in the scan path. When this frontal signal was unilaterally lesioned the scan path exhibited symptoms of hemineglect similar to those observed following parietal lesion; an example is shown in figure 7a. However, attention was sometimes attracted to the left hemifield and neglect was less severe than that following parietal lesion, as shown in figure 7b. Note that the frontal and parietal representations have differing frames of reference: scene(or body)-based and retinotopic respectively, but both lesions led to neglect during covert and overt attentional scanning. Unlike the effect shown in figure 6 for parietal lesion, **our frontal lesion did not affect extrastriate responses**.

When the frontal bias was completely lesioned, IOR was impaired, locations were repeatedly revisited and there was difficulty re-orienting attention so that the scan path failed to fully explore the scene. Figures 7c shows a typical scan path demonstrating this perseverance in one area.

### Re-visiting Locations in the Scan Path

In addition to frontal lesion resulting in difficulties re-orienting attention and perseverance, we found that unilateral parietal lesion also resulted in increased rates of re-visiting the same locations during the scan path. A comparison of re-fixation rates under the lesion conditions and the control condition is shown in figure 7d. Under conditions of unilateral parietal lesion, the increase in re-fixation rate is not as large as the 13 times increase observed in a patient with neglect due to right inferior parietal lobe infarct [1]. This likely reflects the fact that not all functions of the parietal cortex are modelled here. Also, differences could be due to the extent of the patient's lesion, and the fact that we do not model frontal cortex regions that might provide executive strategies to guide the search scan path. Re-fixation is most prominent under conditions of frontal lesion in our model, particularly when the frontal signal is completely lesioned. Whilst our modelling of frontal cortex is limited in nature, the bias signal modelled here is specific in that it represents a signal from an area representing spatial locations encoded in a scene-based co-ordinate system. This signal biases attentional capture processing in retinotopic parietal cortex. Lesion to this frontal bias in our model resulted in very irratic scan paths. Irratic scan paths with increased re-fixation have been observed in an orbitofrontal patient [7].

**Figure 8:**
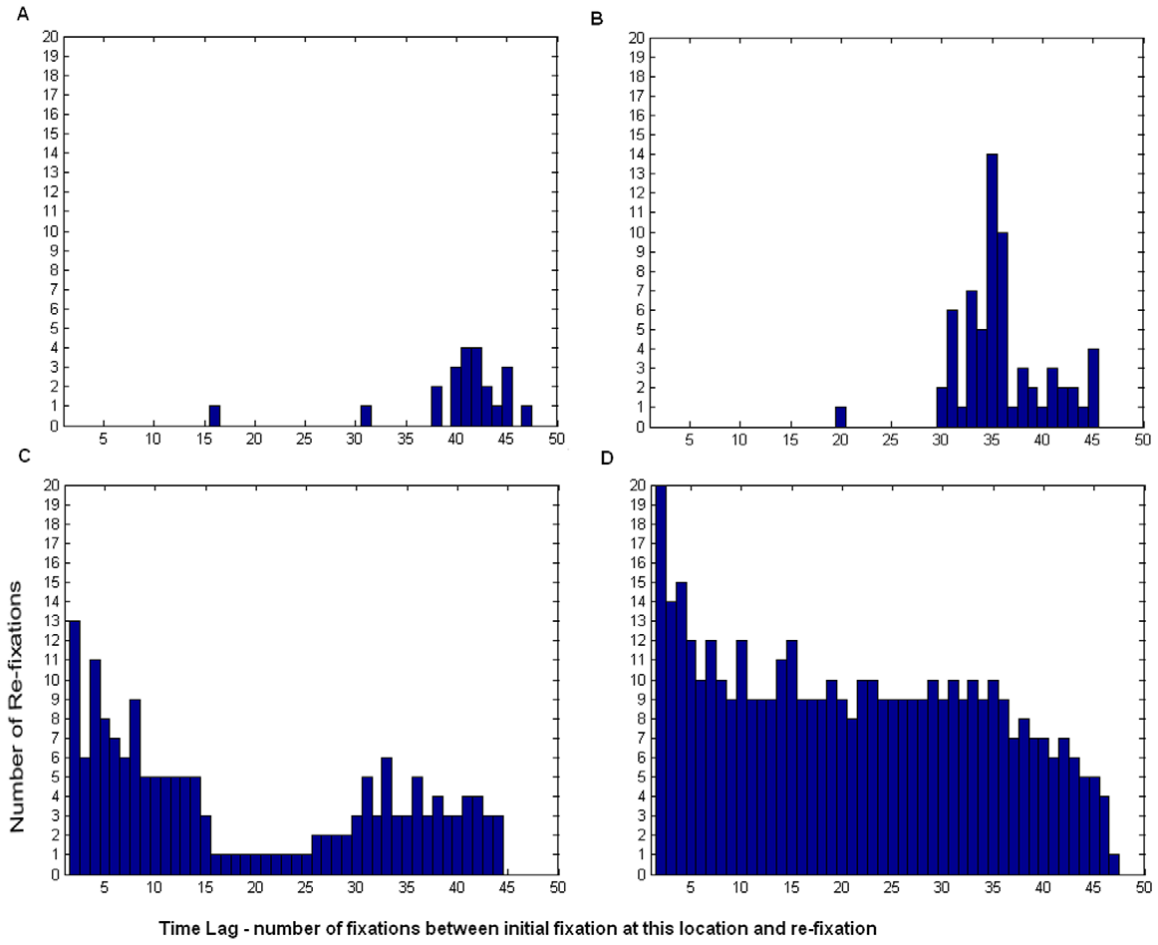
Re-visiting Locations in the Scan Path Over Time. Shows the numbers of re-fixations (or simply re-visiting the same location during covert attentional scanning) that occur at each time lag since that location was first visited under (a) normal conditions, (b) unilateral parietal lesion, (c) unilateral frontal lesion, (d) bilateral frontal lesion. Similar to that reported by Mannan et al. [1], [5], immediate re-fixations i.e. those occurring at the subsequent fixation (time lag of 1) have been removed. Whereas parietal lesion causes an increase in re-fixation only at longer time lags, frontal lesion causes re-fixation increases and short and longer time lags.

Whilst parietal patients have a tendency to re-fixate more with time since first fixating the location, this increase in re-fixation rate with time is not a feature of frontal lesion patient behaviour [1]. We find a similar difference here. Figure 8 shows the number of re-fixations that were made at each time lag since first fixating the location (in terms of numbers of subsequent fixations). Under normal conditions there is a slight tendency to re-fixate locations as more time has passed since they were first inspected. Under parietal lesion conditions, this tendency to re-fixate increases. Importantly, the parietal-lesioned model is more likely to re-fixate locations at larger time lags since first fixation. In contrast, with a frontal lesion, re-fixation is not dependent on time since first fixation.

## Discussion

We extended our model of visual attention to investigate lesions that lead to symptoms of visual neglect. Our modelling approach is based broadly on the theory of biased competition [40] and seminal computational modelling work in the field of visual attention by Deco [23] and Usher and Niebur [41]. Biased competition is based on the idea of competition between neurons throughout the visual hierarchy, a concept that was presented earlier by Tsotsos in his ‘selective tuning’ model [42]. Biased competition has become a very influential and predominant theory in the field of visual attention. In both Deco's models and ours, a dynamic system encompasses biased competitive interactions between neurons within the system [11].

Under conditions of unilateral parietal lesion in our computational model, simulated scan path behaviour mimicked that seen in neurological case studies of visual neglect, where scan paths are attracted to the ipsilesional hemifield and avoid the contralesional hemifield. We find that parietal lesion results in neglect of the ipsilateral hemifield under conditions of both covert and overt attentional scanning. Patients also tend to fail to remember previously searched locations so that they make repeated saccades back to them, particularly as time since first fixating the location increases [1], [5]. Similarly, simulated pathological scan paths exhibited higher than normal re-visiting of locations, although not quite as high as those in patients. Differences might be due to the extent of modelled anatomy compared to the extent of the patient's lesion. Consistent with patient behaviour, re-fixations tended to be made at larger time lags since first fixating a location.

Scan paths from our simulations of overt attention tended to be attracted to and unable to re-orient away from the right edge of the scene. This could be due to the artificial extent of our scenes compared to the continuous real world. However, this could also reflect real visual neglect deficiencies when viewing a scene of a fixed size. Such difficulties could be partially overcome by utilising cognitive control strategies to reset fixation back into the scene. For our simulations we applied a “top-down” strategy where a small leftward saccade was made to shift fixation back into the scene in the situation where fixation lands at the rightmost border of the scene (note, although reports of laterality bias in patients' saccades vary, some show as many leftward as rightward saccades in the patient's normal hemifield [1], [39]). Such a bias could represent a “top-down” bias from frontal cortex and we predict that parietal patients with intact frontal cortex may be better able to use such relocation strategies than patients whose lesions involve executive areas of frontal cortex that are involved in search planning (the exact location of these areas has yet to be established).

Our frontal novelty representation is based on a scene (or body) -based frame of reference. We lesioned this novelty bias to examine the effect of damage to a non-retinotopic frontal area, specifically a region such as orbitofrontal cortex, which is involved in search strategies based on novelty and/or reward [7]. In patients, scanning deficits have been observed in various frames of reference, including body-centred [43]. We have not distinguished between scene-based and body-centred representations since we do not model a body moving in space. It is possible that the correlates of this “novelty map” exist somewhere other than frontal cortex, for example in another region of parietal cortex. We found that unilateral lesion of our scene-based frontal module resulted in neglect of the contralesional hemifield, though slightly less severe than that following parietal lesion. Hence, we find that our model neglects the contralesional hemifield following unilateral parietal or frontal lesion. However, neglect was more severe following retinotopic parietal lesion and attention was sometimes attracted to the contralesional hemifield with a scene-based frontal lesion.

Whilst we observed deficits in scan path behaviours, we did not find differences in levels of extrastriate activity following lesion to our frontal bias. This suggests that these effects in neglect are dissociable. Neuroimaging studies comparing levels of activity in extrastriate cortex for consciously perceived and neglected stimuli have so far focussed on patients with parietal lesion. Studies of neural activity and connectivity in frontal neglect patients, using functional MRI, event-related potentials and effective connectivity, would be useful. We predict that neuroimaging of extrastriate cortex activity under conditions of frontal lesion neglect would not show the difference in level of activity observed under parietal lesion neglect for consciously perceived versus neglected stimuli.

We were not attempting to model all functions of the frontal cortex that relate to visual search, but modelled a very specific signal from an area representing spatial locations encoded in a scene-based co-ordinate system that biases attentional capture processing in retinotopic parietal cortex. Lesions, particularly a bilateral lesion, to this frontal bias resulted in very restricted scan paths with high levels of re-fixation. Hodgson et al. [7] and Na et al. [6] have noted that frontal lesion patients exhibit motor perseveration and re-visit locations more often than controls. Na et al. [6] found that neglect patients with frontal damage (compared to those with more posterior lesions alone) are more likely to have perseveration, making multiple visible marks on lines during standard paper-and-pen cancellation tasks. An orbitofrontal patient (without neglect) examined by Hodgson et al. [7] had erratic search scan paths with high re-fixation rates. Therefore, these anterior lesions tend to be associated with re-fixation [7] and produce more motor perseveration [6] rather than increasing severity of neglect. We find qualitatively similar results in simulations here: lesion of the frontal bias leads to a failure of IOR so that locations in the scene are often revisited, there is a tendency to perseverate and scan paths are irratic. Other frontal functions, not modelled here, may provide further executive search strategy that control scan path behaviour.

The nature of re-fixation differed depending on locus of lesion. For our parietal lesion simulations, there was a tendency to re-visit locations after some time had elapsed since first visiting that location. However, under frontal lesion, locations were often re-visited at short time lags as well as at longer time lags. Hence, frontal lesion in our model results in a break-down in IOR and an inability to remember which locations the scan path has revisited whereas parietal lesion increases re-fixation rates but this principally affects fixations at longer time lags from the time the location was first fixated. These effects mirrored behaviours seen in patients [1]. In our model this difference is due to the fact that, when the frontal bias is ineffective, competition in the parietal module is not biased by novelty and attention is guided purely by the output of competition in the model's ventral stream, which is driven by bottom-up stimulus factors combined with the top-down attention modulation related to the task (search target object). Hence, each fixation at the same location results in a very similar outcome in the parietal module, because inputs are the same each time. The competition between locations in the parietal module determines which location captures attention next. Thus, for any particular search task (e.g. find the red vertical bar), each time fixation is at a particular location and the retinal window contains the same surrounding stimuli at that location, the same stimulus location will win the competition to attract attention. This results in perseveration and attention continually being drawn to certain highly attractive (in the sense of behavioural salience, i.e. bottom-up saliency combined with top-down target object-based attention) locations. These locations are not inhibited over time and there is a tendency to select the same location again and again, even from the start of the scan path, and be unable to fully explore the scene. However, when the frontal signal is effective in biasing competition within the parietal module, each time fixation arrives at a particular location the novelty of surrounding stimuli (given by scan path “history”, and recorded here in our scene-based novelty map) will be different and will lead to a different outcome, with more novel locations being favoured in the parietal competition. Hence, fixation will be driven towards new locations and inhibited from locations already inspected. In contrast, parietal lesion results in locations represented in the damaged hemisphere, i.e. the neglected hemifield, being prevented from winning the competition to attract attention. Locations in the intact hemifield compete normally to attract attention under the influence of the novelty bias so that more novel locations in the intact hemifield will be favoured. As more locations in the intact hemifield are inspected, overall novelty becomes lower and the novelty bias becomes less effective. The hemifield damage in the parietal module makes it unlikely for novel locations in the neglected half of the original image to be inspected and hence there is a tendency, over time, for locations in the non-neglected half of the image to be re-inspected.

Our simulation results differ from previous modelling contributions in that our model is based on a detailed level of neurophysiological modelling in which behaviours of individual model neuron capture effects observed in single unit recordings [11] and the systems level visual search behaviours are based upon these effects [8]. Some models of neglect, for example the Selective Attention for Identification model (SAIM: [35]) and the MORSEL model [36] are less neurobiologically-constrained. In the SAIM model inputs from a feature detecting ‘content’ network are gated by a ‘selection network’, which is linked to the pulvinar and/or parietal cortex but does not specifically model neuronal response properties from these areas. Hence, the level of abstraction is higher than that in our model or that of Deco and Rolls [34] where neuronal behaviours are based closely upon neurophysiology. In SAIM the selection network determines which inputs from the content network are mapped into the Focus of Attention (FOA) and lesions to the selection network result in symptoms of visual neglect. If the selection network is lesioned vertically one side of the visual field is affected (hemifield neglect), whereas a horizontal lesion affects one side of the FOA such that, when a single object is present within the FOA, the left-side is neglected producing a more object-based effect. Simulations in SAIM use very small images of 7×7 pixels (contrasted, for example, with our scenes which are at least 880×880 pixel complex scenes) containing one or two objects and do not produce overt eye movements. However, the architecture provides a very interesting insight into both spatial and object-based neglect at a fairly high level of abstraction that could be expanded upon to further elucidate the neural underpinnings of the theory. Our model operates at a level of abstraction that is closer to the neurophysiology in that it also captures neuronal behaviours at the individual cell level within the cortical areas that are modelled [11]. A model of basis functions reflecting different coordinate systems in the parietal function [44] provides insight into parietal encoding and function and can simulate neglect when lesioned. However, this model does not include interactions with other areas of cortex. Most closely allied to our model, Deco and Rolls [34] used a simplified version of Deco's visual attention model [23], [24], [45], to simulate parietal lesions leading to visual neglect behaviour during visual search. Their model is based on neurophysiology and includes V1 and a posterior parietal (PP) module, which are connected by Gaussian connection weights, and an IT module connected to V1. For the lesion simulations, local lateral connections were introduced in the V1 and PP modules to provide inhibitory input from surrounding locations. Object-based neglect of the left side of individual objects (or two objects connected as a single entity) was simulated using a gradient of impairment in the parietal module. These simulations provided valuable insight because the neurological symptoms of object-based neglect were accurately simulated by a model that is constrained by neurobiology. Neglect is determined in this model by a very small difference in activity in the PP module. It is not clear whether this threshold would generalize or is to the tuned to the specific stimulus configuration. This differs from our simulations in which lesion of the parietal module led to reduced activity in the extrastriate ventral stream, which has been directly linked to differences in conscious perception [21], [22], [46], [47].

The anatomy of neglect is controversial and the syndrome is heterogeneous, with a range of spatial and non-spatial cognitive deficits being exhibited by patients [48]. Our parietal module encodes stimuli in a retinotopic manner, similar for example to those representations found in monkey inferior parietal sulcus in the lateral intraparietal region [28]. Human homologues of monkey parietal cortex are not clear and there appear to be regions in the human inferior parietal lobe that do not have a clear homology to monkey posterior parietal cortex [49]. However, like monkey parietal cortex [50], the organisation of human parietal cortex involves many differing frames of reference and coordinate systems, including a retinotopic reference frame [51]. Computational modelling of non-spatial forms of neglect and more complex forms of spatial neglect could benefit from a more detailed model of human parietal cortex function in which the nature of representation in different regions is elaborated, and methods of information transfer between different coordinate schemes and interfaces with the ventral stream are established. Further development of such models will be possible as more complete mapping of human parietal function, and identification of its monkey homologues, emerges from human functional neuroimaging and diffusion tensor imaging.

Following unilateral lesion to the parietal module, our simulations showed activity for neglected stimuli was lower in V4 than that for consciously perceived stimuli. Activity in the human ventral visual stream (striate and extrastriate cortex) has been linked with conscious awareness of stimuli. Event-related potential and functional MRI studies [20], [22], [47] show that activity is present in striate and extrastriate ventral stream regions for both consciously perceived stimuli and stimuli that are not consciously perceived in neglect cases following of parietal lesion. However, activity is reduced in the situation where the stimulus is not consciously perceived [21], [22], [47]. Hence, reduced activity in ventral stream areas in response to neglected stimuli, as compared to normally perceived stimuli, may be the neural correlate of unconscious versus conscious perception in parietal neglect [21], [22], [47]. The effect that we observed here appears to be a possible computational correlate of these differences in levels of neural activity observed in functional MRI and event-related potential recordings. In our model, these differences in ventral stream activity result from the connection between the parietal module and area V4. Hence, our model simulations lead to the prediction that dorsal-ventral cross-stream connections may be an important factor in perceptual deficits in neglect. Further, Vuilleumier et al. [47] used correlations in functional MRI BOLD activity to examine the effective connectivity between visual regions and parietal and frontal areas in neglect patients. They found that connectivity increased in conditions of conscious versus unconscious perception. In our model, the connection between the parietal cortex and the ventral stream is more active in regions that are not neglected and this leads to higher levels of activity in V4 for normally perceived stimuli than neglected stimuli. On the basis of our model, and the evidence of effective connectivity [47], we suggest that interaction between the ventral and dorsal visual processing streams, i.e. from parietal cortex to extrastriate cortex, is one important component in the explanation of perceptual deficits in parietal lesion patients and of conscious perception in general.

## Supporting Information

Appendix S1 Appendix containing the formal definition of the model.
(0.09 MB DOC)
