# Supplementary material for: Modelling Visual Neglect: Computational Insights into Conscious Perception
Source: PLoS One. 2010 Jun 15;5(6):e11128. doi: 10.1371/journal.pone.0011128 (PMC2886104; doi:10.1371/journal.pone.0011128)
Supplement: Appendix S1 — Appendix containing the formal definition of the model. (0.09 MB DOC) [file pone.0011128.s001.doc]

**Appendix S1 – Formal description of model**

For reasons of simplicity description of the retina feature detection processes are not included here but, for a complete model description, the reader is referred to [1,2,3]. Modules representing areas V4, IT and LIP interact dynamically to produce the attentional effects within the system. The retina and V1 do not form part of the dynamic portion of the system but act as feature detectors. The number of units in V4 and the parietal module scales with the chosen size of the retina (see [1]) but typically comprise an array of 20 x 20 units in the parietal module and in each feature layer in V4. Area V1 consists of a unit for each feature detected at every position in the original image, which is typically of size 881x881 pixels for the simulations here.

**S1-1 V1**

The V1 module consists of K + C neurons at each location in the original image, so that neurons detect K orientations and C colours. Only those V1 outputs relating to the current retinal image are forwarded to V4 during the dynamic active vision processing. The size of filters used in V1 determines the ratio of pixels to degrees of visual angle so that V1 receptive fields cover approximately 1° of visual angle [4].

**S1-1.1 Form Processing in V1**

## For orientation detection, V1 simple and complex cells are modelled as described by Grossberg & Raizada [5], with the distinction that two spatial resolutions are calculated here. Simple cells detect oriented edges using a difference-of-offset-Gaussian (DOOG) kernel:

The right and left-hand kernels of the simple cells are given by:

R_rijk_ = Σ_pq_([*u*^+^_pq_]_+_ - [*u*^-^_pq_]_+_) [D^(lk)^_pqij_]_+_ [6]

L_rijk_ = Σ_pq_([*u*^-^_pq_]_+_ - [*u*^+^_pq_]_+_) [-D^(lk)^_pqij_]_+_ [7]

where:

*u*^+^ and *u*^-^ are the outputs of the retinal broadband cells [3]

[x]_+_ signifies half-wave rectification, i.e. [x]_+ =_ x if x$\geq0$; 0 otherwise

## and the oriented DOOG filter D^(lk)^_pqij_ is given by:

D^(lk)^_pqij_ = G_pq_(i - δcosθ, j - δsinθ, σ_2_) - G_pq_(i + δcosθ, j + δsinθ, σ_2_) [8]

where:

δ = σ_2_/2 and θ = π(k-1)/K, where k ranges from 1 to 2K, K being the total number of orientations (2 is used here).

σ_2_ is the width parameter for the DOOG filter, set as below

r is the spatial frequency octave (i.e. spatial resolution), such that

r = 1 and σ_2_ = 1.2 for high resolution processing, used in the parvocellular pathway, which forms the remainder of the model;

r = 2 and σ_2_ = 2.2 for low resolution processing, used in the magnocellular (or sub-cortical) pathway for scaling the AW

The direction-of-contrast sensitive simple cell response is given by:

S_rijk_ = γ[R_rijk_ + L_rijk_ - |R_rijk_ – L_rijk_|]_+_ [9]

γ is set to10

The complex cell response is invariant to direction of contrast and is given by:

I_rijk_ = S_rijk_ + S_rij(k+K)_ where k ranges from 1 to K [10]

The value of the complex cells, I_rijk_, over the area of the current retinal image, is input to V4.

**S1-1.2 Colour Processing in V1**

The outputs of LGN concentric single-opponent cells (simplified to be the retinal cells here) are combined in the cortex in the double-opponent cells concentrated in the blob zones of layers 2 and 3 of V1, which form part of the parvocellular system. The outputs of blob cells are transmitted to the thin stripes of V2 and from there to colour-specific neurons in V4. For simplicity, V2 is not included in this model.

Double-opponent cells have a centre-surround antagonism and combine inputs from different single-opponent cells as follows:

Red on-centre portion: [11]

$$\omega_{ij}^{redON}=\sum_{pq} G_{pq}(i,j,\sigma_{1})\nu_{ij}^{redON}+\sum_{pq} G_{pq}(i,j,\sigma_{1})\nu_{ij}^{greenOFF}-\sum_{pq} G_{pq}(i,j,\sigma_{1})\nu_{ij}^{redOFF}-\sum_{pq} G_{pq}(i,j,\sigma_{1})\nu_{ij}^{greenON}$$

Red off-surround portion: [12]

$$\omega_{ij}^{redOFF}=\sum_{pq} G_{pq}(i,j,\sigma_{2})\nu_{ij}^{redOFF}+\sum_{pq} G_{pq}(i,j,\sigma_{2})\nu_{ij}^{greenON}-\sum_{pq} G_{pq}(i,j,\sigma_{2})\nu_{ij}^{redON}-\sum_{pq} G_{pq}(i,j,\sigma_{2})\nu_{ij}^{greenOFF}$$

Green on-centre portion: [13]

$$\omega_{ij}^{greenON}=\sum_{pq} G_{pq}(i,j,\sigma_{1})\nu_{ij}^{greenON}+\sum_{pq} G_{pq}(i,j,\sigma_{1})\nu_{ij}^{redOFF}-\sum_{pq} G_{pq}(i,j,\sigma_{1})\nu_{ij}^{greenOFF}-\sum_{pq} G_{pq}(i,j,\sigma_{1})\nu_{ij}^{redON}$$

Green off-surround portion: [14]

$$\omega_{ij}^{greenOFF}=\sum_{pq} G_{pq}(i,j,\sigma_{2})\nu_{ij}^{greenOFF}+\sum_{pq} G_{pq}(i,j,\sigma_{2})\nu_{ij}^{redON}-\sum_{pq} G_{pq}(i,j,\sigma_{2})\nu_{ij}^{greenON}-\sum_{pq} G_{pq}(i,j,\sigma_{2})\nu_{ij}^{redOFF}$$

where:

*ν* are the outputs of the retinal concentric single opponent cells [3]

σ_1_=1.2, σ_2_=1.5

The complete red-selective blob cell is given by:

$I_{ijc^{1}}=\gamma\left[\omega_{ij}^{redON}-\omega_{ij}^{redOFF}\right]_{+}$ [15]

The complete green-selective blob cell is given by:

$I_{ijc^{2}}=\gamma\left[\omega_{ij}^{greenON}-\omega_{ij}^{greenOFF}\right]_{+}$ [16]

where:

γ = 0.2 This scales the output of V1 blob cells to be consistent with that of the orientation-selective cells

c^1^ = K + 1 This represents the position of the first colour input to V4 (i.e. red)

c^2^ = K + 2 This represents the position of the second colour input to V4 (i.e. green)

The blob cell outputs over the area of the current retinal image are input to V4.

**S1-2 Dynamic Cortical Modules**

The dynamic cortical modules follow a similar approach to that described by Deco [6,7,8] and are modelled using mean field population dynamics [9,10] also used by Usher and Niebur [11]. In this mean field approach average ensemble activity is used to represent populations, or assemblies, of neurons with similar encoding properties. The response function, which transforms current (activity within the assembly) into discharge rate, is given by the following sigmoid function that has a logarithmic singularity [9]:

$F(x)=\frac{1}{T_{r}-\tau\log(1-\frac{1}{\tau x})}$ [17]

where:

T_r_, the absolute refractory time, is set to 1ms

τ, is the membrane time constant (where $\frac{1}{\tau}$determines the cell’s firing threshold).

**S1-2.1 V4**

V4 consists of a three dimensional matrix of pyramidal cell assemblies. The first two dimensions represent the retinotopic arrangement and the other represents the feature types. In the latter dimension, there are K + C layers of cell assemblies: the K layers each selective for an orientation, the C layers each selective for a particular colour. Two orientations (vertical and horizontal) and two colours (red and green) are normally used. Two sets of inhibitory interneuron pools exist: One set mediates competition between orientations and the other mediates competition between colours. V4 receives convergent input from V1 over the area of its receptive field with a latency of 60ms to reflect normal response latencies [12]. In order to simulate the normalisation of inputs occurring during retinal, LGN and V1 processing, the V1 inputs to V4 are normalised by passing the convergent inputs to each V4 assembly through the response function at equation 17 with its threshold set to a value equivalent to an input activity for approximately half a stimulus within its receptive field.

**S1-2.1.1 Form processing in V4**

The output from the V1 simple cell process, I_ijk_, for each position (i,j) at orientation k, provides the bottom-up input to orientation selective pyramidal assemblies in V4 that evolve according to the following dynamics:

$\begin{matrix} \tau_{1}\frac{\delta}{\delta t}W_{ijk}(t)=-W_{ijk}(t)+ \alpha F\left(W_{ijk}(t)\right)-\beta F\left(W_{ij}^{IK}(t)\right)+\chi\sum_{pq} I_{pqk}(t)+ \\ +\gamma Y_{ij}(t)+ \eta\sum_{m} B_{W_{ijk}X_{m}}.F(X_{m}(t))+I_{0}+\nu\end{matrix}$ [18]

## where:

τ_1_ is set to 20ms

α is the weight of excitatory input from other cells in the pool, set to 0.95

β is the weight of inhibitory interneurons input, set to 10

I_pqk_ is the input from the V1 simple cell edge detection process at all positions within the V4 receptive field area (p,q), and of preferred orientation k

χ is the weight of V1 inputs, set to 4

Y_ij_ is the input from the parietal module, reciprocally connected to V4

γ is the weight of the parietal module inputs, set to 3

X_m_ is the feedback from IT cell populations via weight $B_{W_{ijk}X_{m}}$, described later

η is the parameter representing the strength of object-related feedback from IT; set to 5

I_0_ is a background current injected in the pool, set to 0.25

ν is additive noise, which is randomly selected from a uniform distribution on the interval (0,0.1)

The dynamic behaviour of the associated inhibitory pool for orientation-selective cell assemblies in V4 is given by:

$\tau_{1}\frac{\delta}{\delta t}W_{ij}^{IK}(t)=-W_{ijk}^{IK}(t)+ \lambda\sum_{k} F\left(W_{ijk}(t)\right)-\mu F\left(W_{ij}^{IK}(t)\right)$ [19]

where:

λ is the weight of pyramidal cell assembly input, set to 1

μ is the weight of inhibitory interneuron input, set to 1

Over time, this results in local competition between different orientation selective cell assemblies.

# **S1-2.1.2 Colour processing in V4**

The output from the V1 simple cell process, I_ijc_, for each position (i,j) and colour c, provides the bottom-up input to colour selective pyramidal assemblies in V4 that evolve according to the following dynamics:

$\begin{matrix} \tau_{1}\frac{\delta}{\delta t}W_{ijc}(t)=-W_{ijc}+ \alpha F\left(W_{ijc}(t)\right)-\beta F\left(W_{ij}^{IC}(t)\right)+\chi\sum_{pq} I_{pqc}(t)+\gamma Y_{ij}(t) \\ + \eta\sum_{m} B_{W_{lijc}X_{m}}.F(X_{m}(t))+I_{0}+\nu\end{matrix}$ [20]

where:

I_pqc_ is the input from the V1 blob cells at all positions within the V4 receptive field area (p,q), and of preferred colour c

X_m_ is the feedback from IT cell populations via weight$B_{W_{ijc}X_{m}}$, described later

The remaining terms are the same as those in equation 18.

The dynamic behaviour of the associated inhibitory pool for colour-selective cell assemblies in V4 is given by:

$\tau_{1}\frac{\delta}{\delta t}W_{ij}^{IC}(t)=-W_{ijc}^{IC}(t)+ \lambda\sum_{c} F\left(W_{ijc}(t)\right)-\mu F\left(W_{ij}^{IC}(t)\right)$ [21]

Parameters take the same values as those in equation 19.

Over time, this results in local competition between different colour selective cell assemblies.

**S1-2.2 IT**

The model IT encodes all possible objects and receives feedforward feature inputs from V4 with a latency of 80ms to reflect normal response latencies [4]. V4 inputs to IT are normalised by dividing the total input to each IT assembly by the total number of active (i.e. non-zero) inputs. IT also feeds back an object bias to V4. The strength of these connections is given by the following weights, which are set to –1 or 0, as appropriate, for inhibitory feedback to features not belonging to this object.

V4 Cell Assemblies to IT (Feedforward)

$A_{X_{m}W_{ijz}}$ [22]

IT to V4 Cell Assemblies (Feedback)

$B_{W_{ijz}X_{m}}$ [23]

# where z indicates orientation, k, or colour, c

The pyramidal cell assemblies in IT evolve according to the following dynamics:

$$\begin{matrix} \tau_{1}\frac{\delta}{\delta t}X_{m}(t)=-X_{m}(t)+ \alpha F\left(X_{m}(t)\right)-\beta F\left(X^{I}(t)\right)+ \chi\sum_{ijk} A_{X_{m}W_{ijk}}.F\left(W_{ijk}(t)\right)+\chi\sum_{ijc} A_{X_{m}W_{ijc}}.F\left(W_{ijc}(t)\right) \\ +\gamma P_{M}^{v}(t)+\gamma P_{M}^{vBackground}(t)+I_{0}+\nu\end{matrix}$$

[24]

where:

β is the weight of inhibitory interneuron input, set to 0.01

W_ijk_ is the feedforward input from V4 relating to orientation information, via weight $A_{X_{m}W_{ijk}}$

W_ijc_ is the feedforward input from V4 relating to colour information, via weight $A_{X_{m}W_{ijc}}$

- is the weight of V4 inputs, set to 2.5

γ is the weight of the object-related bias from prefrontal cortex, set to 1.2

$P_{M}^{v}$ is the object-related feedback current from ventrolateral prefrontal cortex, injected directly into this pool

This feedback is sigmoidal over time as follows:

For the target object:

$P_{M}^{v}$= 0

Other objects receive inhibitory feedback as follows:

$P_{M}^{v}$ = -1/(1+exp(τ_sig_-t)) [25]

where t = time (in milliseconds) and

τ_sig_ is the point in time where the sigmoid reaches half its peak value: set to 150ms in order to accurately replicate the time course of attentional effects in individual cells [3].

The remaining terms and parameters are evident from previous equations.

The dynamic behaviour of the associated inhibitory pool in IT, providing competition between objects, is given by:

$\tau_{1}\frac{\delta}{\delta t}X^{I}(t)=-X^{I}(t)+ \lambda\sum_{m} F\left(X_{m}(t)\right)-\mu F\left(X^{I}(t)\right)$ [26]

where:

λ is the weight of pyramidal cell assembly input, set to 3

- is the weight of inhibitory interneuron input, set to 1

### S1-3 References

1. Lanyon LJ, Denham SL (2004) A model of active visual search with object-based attention guiding scan paths. Neural Netw 17: 873-897.

2. Lanyon LJ, Denham SL (2004) A biased competition computational model of spatial and object-based attention mediating active visual search. Neurocomputing 58-60: 655-662.

3. Lanyon LJ, Denham SL (2009) Modelling attention in individual cells leads to a system with realistic saccade behaviours. Cogn Neurodyn 3: 223-242.

4. Wallis G, Rolls ET (1997) Invariant face and object recognition in the visual system. Prog Neurobiol 51: 167-194.

5. Grossberg S, Raizada RD (2000) Contrast-sensitive perceptual grouping and object-based attention in the laminar circuits of primary visual cortex. Vision Res 40: 1413-1432.

6. Deco G (2001) Biased competition mechanisms for visual attention in a multimodular neurodynamical system. In: Wermter S, Austin, J, Willshaw, D, editor. Emergent Neural Computational Architectures Based on Neuroscience: Towards Neuroscience-Inspired Computing Heidelberg: Springer-Verlag. pp. 114-126.

7. Deco G, Lee TS (2002) A unified model of spatial and object attention based on inter-cortical biased competition. Neurocomputing 44: 775-781.

8. Rolls ET, Deco G (2002) Computational Neuroscience of Vision. Oxford: Oxford University Press.

9. Gerstner W (2000) Population dynamics of spiking neurons: fast transients, asynchronous states, and locking. Neural Comput 12: 43-89.

10. Wilson HR, Cowan JD (1972) Excitatory and inhibitory interactions in localized populations of model neurons. Biophys J 12: 1-24.

11. Usher M, Niebur E (1996) Modeling the Temporal Dynamics of IT Neurons in Visual Search: A Mechanism for Top-Down Selective Attention. Journal of Cognitive Neuroscience 8: 311-327.

12. Luck SJ, Chelazzi L, Hillyard SA, Desimone R (1997) Neural mechanisms of spatial selective attention in areas V1, V2, and V4 of macaque visual cortex. J Neurophysiol 77: 24-42.
